# Increased development of radiographic hip osteoarthritis in individuals with high bone mass: a prospective cohort study

**DOI:** 10.1186/s13075-020-02371-0

**Published:** 2021-01-06

**Authors:** April Hartley, Sarah A. Hardcastle, Monika Frysz, Jon Parkinson, Lavinia Paternoster, Eugene McCloskey, Kenneth E. S. Poole, Muhammad K. Javaid, Mo Aye, Katie Moss, Martin Williams, Jon H. Tobias, Celia L. Gregson

**Affiliations:** 1 Musculoskeletal Research Unit, Translational Health Sciences, Bristol Medical School, University of Bristol, Bristol, UK; 2 MRC Integrative Epidemiology Unit, Population Health Sciences, Bristol Medical School, University of Bristol, Bristol, UK; 3 Royal National Hospital for Rheumatic Diseases, Royal United Hospitals Bath NHS Foundation Trust, Bath, UK; 4 Division of Informatics, Imaging & Data Sciences, Faculty of Medical and Human Sciences, University of Manchester, Manchester, UK; 5 Academic Unit of Bone Metabolism, Department of Oncology and Metabolism, The Mellanby Centre For Bone Research, University of Sheffield, Sheffield, UK; 6 Centre for Metabolic Diseases, University of Sheffield Medical School, Sheffield, UK; 7 Centre for Integrated Research into Musculoskeletal Ageing, University of Sheffield Medical School, Sheffield, UK; 8 Cambridge NIHR Biomedical Research Centre and the Wellcome Trust Clinical Research Facility, Cambridge, UK; 9 Nuffield Department of Orthopaedics, Rheumatology and Musculoskeletal Sciences, University of Oxford, Oxford, UK; 10 Department of Diabetes, Endocrinology and Metabolism, Hull and East Yorkshire Hospitals NHS Trust, Hull, UK; 11 Centre for Rheumatology, St George’s Hospital, St George’s Healthcare NHS Trust, London, UK; 12 Department of Radiology, Southmead Hospital, North Bristol NHS Trust, Bristol, UK

**Keywords:** Hip osteoarthritis, Progression, High bone mass, BMD, WOMAC

## Abstract

**Background:**

Individuals with high bone mass (HBM) have a greater odds of prevalent radiographic hip osteoarthritis (OA), reflecting an association with bone-forming OA sub-phenotypes (e.g. osteophytosis, subchondral sclerosis). As the role of bone mineral density (BMD) in hip OA progression is unclear, we aimed to determine if individuals with HBM have increased incidence and/or progression of bone-forming OA sub-phenotypes.

**Methods:**

We analysed an adult cohort with and without HBM (L1 and/or total hip BMD *Z*-score > + 3.2) with pelvic radiographs collected at baseline and 8-year follow-up. Sub-phenotypes were graded using the OARSI atlas. Superior/inferior acetabular/femoral osteophyte and medial/superior joint space narrowing (JSN) grades were summed and Δosteophyte and ΔJSN derived. Pain and functional limitations were quantified using the WOMAC questionnaire. Associations between HBM status and change in OA sub-phenotypes were determined using multivariable linear/logistic regression, adjusting for age, sex, height, total body fat mass, follow-up time and baseline sub-phenotype grade. Generalised estimating equations accounted for individual-level clustering.

**Results:**

Of 136 individuals, 62% had HBM at baseline, 72% were female and mean (SD) age was 59 (10) years. HBM was positively associated with both Δosteophytes and ΔJSN (adjusted mean grade differences between individuals with and without HBM *β*_osteophyte_ = 0.30 [0.01, 0.58], *p* = 0.019 and *β*_JSN_ = 0.10 [0.01, 0.18], *p* = 0.019). Incident subchondral sclerosis was rare. HBM individuals had higher WOMAC hip functional limitation scores (*β* = 8.3 [0.7, 15.98], *p* = 0.032).

**Conclusions:**

HBM is associated with the worsening of hip osteophytes and JSN over an average of 8 years, as well as increased hip pain and functional limitation.

## Introduction

Osteoarthritis (OA) of the hip is highly prevalent, affecting approximately 1% of the worldwide population, significantly contributing to global disability [1]. Currently, no disease-modifying medications are available; therapy consists of pain management until severity warrants a total hip replacement (THR). Detection of risk factors for hip OA progression offers an opportunity to identify potential targets for the development of therapeutic interventions.

Higher bone mineral density (BMD) has been associated with *prevalent* hip OA in several case-control [2, 3] and population-based studies [4–7]. However, such analyses are complicated as BMD is often measured at the hip and therefore it is hard to determine whether increased BMD is a cause, or feature, of hip OA [4–6, 8]. In men with discordant hip OA, Arokoski et al. found femoral neck (FN)-BMD to be 4% higher in the more severely affected hip, reflecting increased FN volume (measured by MRI) [9]. This may reflect a process known as buttressing, whereby osteophytes extend across the FN to artefactually increase measured BMD [10]. However, Chaganti et al. identified a relationship between total hip (TH) cortical volumetric BMD (vBMD, measurement of which it not artefactually increased by bone size) and hip OA in 3886 men in the Study of Osteoporotic Fractures in Men (MrOS) [5]. Moreover, lumbar spine (LS)-BMD can be artefactually elevated by the presence of spinal osteophytes, a feature of spinal OA [4–6]. However, Nevitt et al. found that the relationship between LS-BMD and severe hip OA persisted despite adjustment for spinal osteophytes [4]. Furthermore, they found a relationship between calcaneal BMD and hip OA in over 4000 women from the Study of Osteoporotic Fractures (SOF), although of lower magnitude than seen for TH-BMD [4].

More recently, in a unique population of individuals with high bone mass (HBM), Hardcastle et al. reported those with HBM to have an increased odds of hip OA, reflecting a greater odds of osteophytosis but not joint space narrowing (JSN) [10]. HBM in index cases was defined as a TH or LS-BMD *Z*-score of at least + 3.2, with a *Z*-score of at least + 1.2 at the other site, identifying a generalised high BMD phenotype [11]. Genetic analysis of HBM individuals suggests that the HBM phenotype is at least in part determined by the polygenic inheritance of multiple BMD-associated loci [12]; thus, the temporal relationship is suggestive of a causal pathway between generalised high BMD and prevalent hip OA.

Fewer longitudinal analyses have addressed the relationship between BMD and the *incidence* and/or *progression* of hip OA. In the Johnston County OA project (JoCo) studying 928 older adults over median 6.5 years, although BMD did not predict incident radiographic hip OA, it was inversely associated with incident *symptomatic* radiographic hip OA [13]. Furthermore, Bergink et al. identified an increased odds of both hip OA incidence and progression in those in the highest quartile of FN-BMD compared to the lowest quartile [14], whilst Hochberg et al. identified a dose-response relationship between both forearm and FN-BMD and the incidence of hip OA in SOF [15].

We aimed to determine the role of high BMD in hip OA by examining whether HBM individuals also have an increased odds of hip OA *incidence* and/or *progression*, using 8-year follow-up data collected in this unique cohort*.* We further aimed to determine the relationship between HBM and clinical features of OA, namely pain and functional limitations.

## Methods

### The high bone mass study

**Fig. 1:**
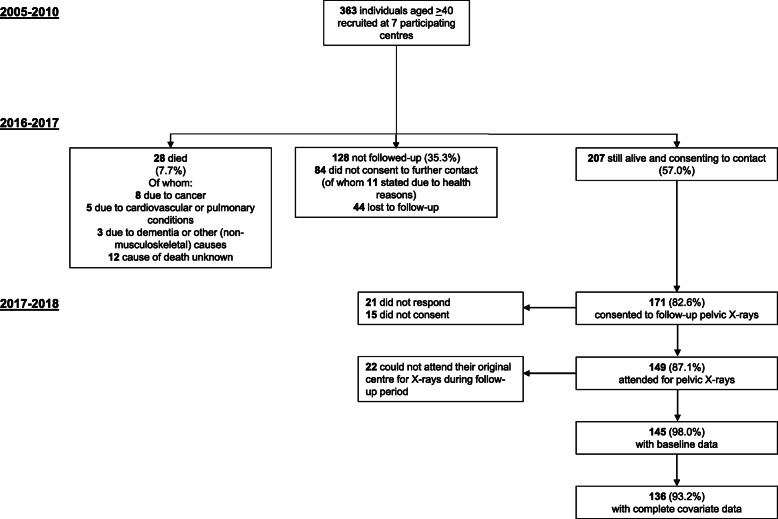
Flowchart detailing the baseline population through 8 years, to derive the follow-up population able to be studied

Participants were recruited as part of the UK-based HBM study. Index cases were initially identified by screening routine clinical National Health Service (NHS) dual-energy X-ray absorptiometry (DXA) databases (254,736 DXA scans from seven UK hospitals) for individuals who had had *T* and/or *Z*-scores > + 4. All 1290 DXA images were inspected by trained clinicians to exclude scans with artefactual elevations of DXA BMD (e.g. degenerative disease, OA, surgical/malignant artefacts). Full details of DXA database screening and participant recruitment have been published [11]. Generalised HBM was defined as a L1 or TH-BMD *Z*-score > + 3.2 with a *Z*-score > + 1.2 at the other skeletal site. A + 3.2 threshold was consistent with the only published precedent for identifying HBM using DXA [16] and most appropriately differentiated generalised HBM from artefact [11]. The use of *Z*-score, rather than *T*-score, limited age bias [11]. Index cases passed on invitations to first-degree relatives and spouses/partners who underwent the same assessments. HBM in spouses was defined as per index cases. In first-degree relatives, HBM was defined as summed L1 plus TH *Z*-score > + 3.2, as this identified relatives with BMD overlapping the index case BMD distribution [11]. Participants who were aged < 18, pregnant or unable to give written informed consent were excluded. Baseline recruitment of 363 adults (237 [65%] with HBM) ran between 2005 and 2010 across seven NHS centres (which participated in follow-up). Two hundred seven (57%) were alive and contactable in 2016; 149 (72%) of whom completed a postal questionnaire and attended for follow-up hip radiographs between 2017 and 2018 (Fig. 1)*.*

### Assessment of BMD

DXA scans were performed of the TH and LS at baseline and, after 8 years follow-up, of the TH, LS and total body (TB) using standard protocols at each assessment centre. All but five (97%) participants re-attended their original centre, limiting measurement error due to differential procedures. DXA scans were performed on Hologic scanners in Bath, Bristol, Sheffield and St George’s London and GE Lunar scanners in Cambridge and Hull. Known differences in calibration exist between Hologic and Lunar [17, 18]. We limited systematic bias by converting TH and LS-BMD measures to standardised BMD (sBMD) [18, 19]. All images were visually inspected for positioning and metal artefacts (e.g. hip prosthesis).

### Assessment of radiographic OA

Standing anteroposterior (AP) pelvic X-rays were performed at baseline and follow-up using standard protocols at each centre. To limit observer bias, all radiographs were pooled for analysis, with the reader blinded to HBM status, demographics and timepoint. Radiographs were graded for semi-quantitative OA sub-phenotypes (osteophytes and JSN, graded 0–3) and subchondral sclerosis (graded as present or absent) using the OARSI atlas [20]. The presence or absence of subchondral cysts was also evaluated. Overall OA was graded using Croft scoring [21]. Generated and derived progression variables are summarised in Table 1. Radiographs, viewed in open source ImageJ software [22], were inspected for poor image quality, rotation and/or tilt. All readings were performed by one assessor (AH) after focussed radiological training with a musculoskeletal radiologist (MW) and rheumatologist (SAH). A random selection of 72 hips (20%) were regraded to determine intra-rater reliability and graded by a second reader (SAH) to determine inter-rater reliability.

**Table 1:** Variables generated by Croft scoring and the OARSI atlas, with additional derived variables

Variable	Grading	Variable used in the analysis
Osteoarthritis (Croft score)	0–5	Progressive OA: Croft score > 3 at baseline and an increase in score at follow-up Incident OA: Croft score < 3 at baseline and > 3 at follow-up
Osteophytes		Change in osteophyte score: sum of all semi-quantitative osteophyte grades at follow-up minus their sum at baseline
*Superior femoral*	0–3	
*inferior femoral*	0–3	
*Superior acetabular*	0–3	
*inferior acetabular*	0, 1	
JSN		Change in JSN score: sum of both superior and medial semi-quantitative JSN grades at follow-up minus this sum at baseline
*Superior*	0–3	
*Medial*	0–3	

*Abbreviations*: *OARSI* Osteoarthritis Research Society International, *JSN* joint space narrowing, *mJSW* medial minimal joint space width

### Assessment of clinical OA

Hip pain and limitation of function were assessed by postal questionnaire at 8-year follow-up. To limit non-response bias, the questionnaire was resent if not returned within 3 weeks. If still unreturned after a further 2 weeks, a reminder telephone call was made. The postal questionnaire included the short version WOMAC function scale [23, 24], which limited participant burden. The pain subscale (five questions relating to pain walking on a flat surface, ascending/descending stairs, at night, sitting or lying and standing upright) and function (seven questions relating to difficulty ascending stairs, rising from sitting, walking on flat, getting in/out of a car, putting on socks/stockings, rising from bed and sitting) each had five possible responses (none, mild, moderate, severe, extreme) scored 0–4, respectively. Missing values for pain or function questions were mean-imputed if a participant was missing one question on the pain scale and < 3 on the function scale. Average scores were calculated for each subscale and scaled to give a score ranging from 0 to 100, with 0 representing no pain or functional limitation [25].

### Covariate data

At baseline, structured interviews and clinical examination determined participant characteristics including age and standing height. Total body fat mass (TBFM) was assessed by TB DXA scan. Baseline menopausal status, alcohol consumption and history of hormone replacement therapy (HRT) use and smoking were determined by researcher-administered questionnaires. Baseline physical activity levels were determined using the International Physical Activity Questionnaire sent by post [26–28]. Menopausal status, history of smoking and highest educational status were determined by postal questionnaire at follow-up.

### Statistical analysis

Associations between HBM status and OA incidence were determined by multivariable logistic regression. We included all hips to increase sample size and thus statistical power, using generalised estimating equations (GEE), which account for correlation between hips from the same individual and produce unbiased estimates in analyses of clustered data [29]. This analysis was restricted to hips with a Croft score < 3 at baseline.

Associations concerning change in osteophytes and JSN (continuous variables) were determined by multivariable GEE linear regression with robust standard errors to account for any non-normal distributions in outcome variables [30, 31]. Betas from analysis of continuous variables represent the difference in mean outcome between those with and without HBM (e.g. a beta of 1 for change in osteophyte score represents a 1-point greater increase in osteophyte score in HBM individuals). Osteophyte and/or JSN scores of 0 at baseline were included in analyses of change in osteophytes and JSN, optimising sample size. Analyses were initially performed unadjusted (model 1) and then adjusted for age, sex and time between radiographs (and baseline sub-phenotype score for continuous outcomes) (model 2). Our previous analyses found HBM to be associated with increased TBFM, with evidence suggesting this is a consequence rather than a cause of HBM [32]. Therefore, adiposity, hypothesised to be on the causal pathway in these analyses, was adjusted for as TBFM in model 3 along with height, to investigate a possible mediating effect of adiposity. Analyses were restricted to individuals with complete data for model 3. Statistical analysis was performed in Stata version 15 (Statacorp, USA) and R version 3.5.1.

### Sensitivity analyses

Joints with THR were excluded from the main analyses; however, as THR may have been performed due to severe OA, those with a baseline Croft score < 3 and THR at follow-up were coded as incident OA cases, if they had stated that their THR was performed due to ‘arthritis’ (*n* = 4). Two individuals without OA at baseline, who had a THR at follow-up due to fracture, were coded as having no incident OA. A person-level analysis, using the sum of the osteophyte and the sum of the JSN scores for the two hips, used GEE to account for correlation within families. Incident OA in person-level analyses represents incident OA in either hip. A model adjusting for metal artefacts on DXA images, analyses removing individuals with DXA positioning errors potentially leading to under-measurement of TBFM (10 hips) and analyses removing individuals who visited a different study site for follow-up (10 hips) were all performed. To check that associations between HBM and change in OA sub-phenotypes were not explained by bone size, we performed an additional analysis adjusting for the FN area (measured at follow-up). Finally, to check if conclusions were valid despite skewed continuous outcomes, all linear analyses were repeated using a Poisson model.

## Results

### Characteristics of the study population

Follow-up radiographic and covariate data were available for 136 individuals, with 62% having HBM (index cases or relatives with HBM). The proportion of individuals with HBM did not differ between the populations with and without follow-up data. Those with follow-up data were younger, were less likely to have had hip OA at baseline, to have ever smoked, to be postmenopausal, but were more physically active (Supplementary Table 1). Mean follow-up time for those with complete data was 8.2 (SD 1.0) years and did not differ between those with and without HBM (Table 2). HBM cases were more commonly female (85 vs 50%), with a trend towards a higher proportion of postmenopausal women. HBM individuals had greater baseline BMD (mean TH-BMD 1.24 vs 0.98 g/cm^2^), BMI (29.8 vs 27.5 kg/m^2^) and TBFM (33.0 vs 29.1 kg) than individuals without HBM (Table 2), consistent with previous observations in this population [11, 32]. Physical activity levels did not differ between HBM individuals and those without HBM.

**Table 2:** Characteristics of the study population, constituting individuals with and without HBM, who were followed up at 8 years

	All, ***N*** = 136	HBM, ***N*** = 86	Relatives without HBM, ***N*** = 50	***p*** value for difference
	***N*** **(%)**			
Female gender	98 (72.1)	73 (84.9)	25 (50.0)	< 0.001
*Postmenopausal*^*b*^	75 (76.5)	59 (80.8)	16 (64.0)	0.087
*Menopause transition during the follow-up period*	11 (11.2)	6 (8.2)	5 (20.0)	0.177
*History of HRT use*^*f*^	49 (50.0)	39 (53.4)	10 (40.0)	0.508
History of smoking^f^	66 (48.9)	42 (49.4)	24 (48.0)	0.874
Physical activity category^b^				
*Low*	14 (10.7)	9 (11.0)	5 (10.2)	
*Medium*	46 (35.1)	26 (31.7)	20 (40.8)	0.567
*High*	71 (54.2)	47 (57.3)	24 (49.0)	
Education category^f^				
*Up to GCSE/O level*	55 (42.0)	42 (50.0)	13 (27.7)	
*A level or equivalent*	26 (19.9)	17 (20.2)	9 (19.2)	0.019
*Degree or equivalent*	50 (38.2)	25 (29.8)	25 (53.2)	
	**Mean (SD)**			
Age, years^b^	59.2 (10.2)	60.2 (9.9)	57.5 (10.6)	0.136
Height, cm^b^	167.8 (9.6)	166.1 (8.4)	170.8 (10.8)	0.005
Weight, kg^b^	81.5 (17.0)	82.1 (16.0)	80.6 (18.7)	0.619
BMI (kg/m^2^)^b^	28.9 (5.5)	29.8 (5.6)	27.5 (5.1)	0.017
TBFM (kg)^f^	31.6 (10.6)	33.0 (10.9)	29.1 (9.5)	0.035
TH-BMD, g/cm^2 b^	1.143 (0.182)	1.242 (0.129)	0.976 (0.131)	< 0.001
L1-BMD, g/cm^2 b^	1.255 (0.215)	1.377 (0.149)	1.049 (0.141)	< 0.001
Follow-up time, years	8.2 (1.0)	8.2 (0.7)	8.2 (1.4)	0.817

*Abbreviations*: *HBM* high bone mass, *HRT* hormone replacement therapy, *BMI* body mass index, *TBFM* total body fat mass, *TH-BMD* total hip bone mineral density

^b^Assessed at baseline

^f^Assessed as follow-up

### Repeatability of radiographic OA variables

Weighted intra-rater kappa statistics for the Croft score and all osteophyte (except inferior acetabular) were > 0.7. The intra-rater reliability kappa for inferior acetabular osteophytes was 0.49, for medial JSN was 0.66 and for superior JSN was 0.49. AH observed no acetabular sclerosis or subchondral cysts. Intra-rater reliability for femoral sclerosis was perfect. Inter-rater weighted kappas for the Croft score and all osteophyte grades (except inferior acetabular) were > 0.6, representing substantial agreement [33]. The inter-rater kappa for inferior acetabular osteophytes was 0.38, with kappas of 0.48 for medial JSN and 0.39 for superior JSN. There was disagreement on the one observed case of femoral sclerosis and the one case of subchondral cysts, so these variables were excluded from analyses.

### HBM and the incidence and progression of overall hip OA

**Fig. 2:**
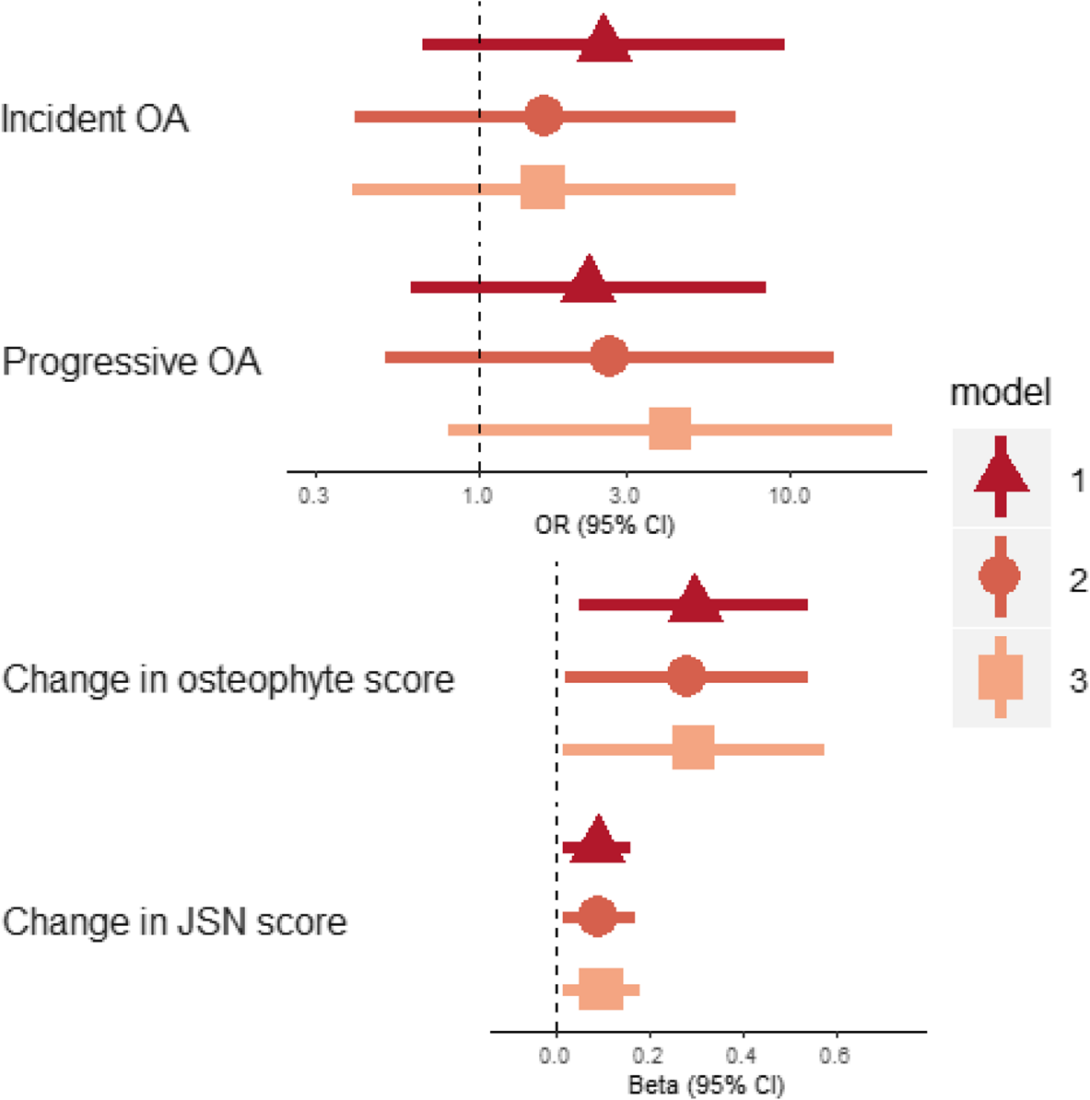
Associations between HBM and incident and progressive OA and change in OA sub-phenotypes. Points for continuous outcomes represent the difference in mean outcome between individuals with and without HBM (for example, a beta of 1 for change in osteophyte score would represent a 1-point greater increase in summed osteophyte score, which is the equivalent of the appearance of one additional osteophyte over 8 years or the increase in the size of an osteophyte already present). Points for binary outcomes represent the odds ratio for individuals with HBM compared to their relatives with normal BMD. Model 1: unadjusted; model 2: adjusted for age, sex and follow-up time (plus baseline score for continuous outcomes); model 3: adjusted for age, sex, follow-up time, height and TBFM (plus baseline score for continuous outcomes). *N*_incident OA_ = 248; *N*_continuous outcomes_ = 263. *Abbreviation*: JSN joint space narrowing

Radiographic hip OA was observed in 7.7% of all 290 hips at baseline and 12.0% at follow-up (Table 3). Of the 257 OA-free hips at baseline, 7.0% developed OA. There was no clear evidence that HBM was associated with an increased risk of overall incident OA measured by Croft score, before (model 1, OR = 2.54 [95%CI 0.66, 9.71], Fig. 2) or after adjustment for age, sex and follow-up time (model 2, 1.65 [0.41, 6.70]). Due to the low baseline prevalence of overall OA defined as Croft score ≥ 3 (i.e. the presence of osteophytes *and* JSN), we were unable to analyse OA progression. Using Croft score > 1 to define OA at baseline, 82 hips had potential to progress, of which 16 had a higher Croft score at follow-up than baseline (12 with HBM). However, no clear association between HBM and overall OA progression was observed (model 3, OR 4.14 [0.81, 21.3], Fig. 2). When combining incident and progressive OA to generate a variable for any incident or progressive hip OA, HBM was still not clearly associated with the overall change in hip OA severity (model 3, OR 1.72 [0.58, 5.11]).

**Table 3:** Prevalence of radiographic and clinical sub-phenotypes of OA in the study population, stratified by HBM status

	**All hips**		**HBM hips**		**Non-HBM hips**	
	**Total** ***N***	***N*** **(%) with sub-phenotype**	**Total** ***N***	***N*** **(%) with sub-phenotype**	**Total** ***N***	***N*** **(%) with sub-phenotype**
OA (Croft > 3)						
*Baseline*	285	22 (7.7)	179	13 (7.3)	106	9 (8.5)
*Follow-up*	275	33 (12.0)	173	24 (13.9)	102	9 (8.8)
*Incident*	257	18 (7.0)	162	15 (9.3)	95	3 (3.2)
*Progressive*	18	5 (27.8)	11	2 (18.2)	7	3 (42.9)
Hip replacement (identified on radiograph)						
*Baseline*	290	5 (1.7)	184	5 (2.7)	106	0
*Follow-up*	290	15 (5.2)	184	11 (6.0)	106	4 (3.8)
*Incident*	285	10 (3.5)	179	6 (3.4)	106	4 (3.8)
Osteophyte score						
*Baseline*	285		179		106	
*0*		203 (71.2)		126 (70.4)		77 (72.6)
*1–4*		75 (26.3)		50 (27.9)		25 (23.6)
*>* *5*		7 (2.5)		5 (2.8)		4 (3.8)
*Follow-up*	275		173		102	
*0*		161 (58.6)		94 (54.3)		67 (65.7)
*1–4*		105 (38.2)		73 (42.2)		32 (31.4)
*>* *5*		9 (3.3)		6 (3.5)		3 (2.9)
*Delta*	275		173		102	
*< 1*		201 (73.1)		121 (69.9)		80 (78.4)
*1*		48 (17.5)		32 (18.5)		16 (15.7)
*> 1*		26 (9.5)		20 (11.6)		6 (5.9)
JSN score						
*Baseline*	285		179		106	
*0*		253 (88.8)		160 (89.4)		93 (87.7)
*1–2*		27 (9.5)		16 (8.9)		11 (10.4)
*>* *3*		5 (1.8)		3 (1.7)		2 (1.9)
*Follow-up*	275		173		102	
*0*		241 (87.6)		149 (86.1)		92 (90.2)
*1–2*		28 (10.2)		20 (11.6)		8 (7.8)
*>* *3*		6 (2.2)		4 (2.3)		2 (2.0)
*Delta*	275		173		102	
*< 1*		261 (94.9)		161 (93.1)		100 (98.0)
*1*		12 (4.4)		10 (5.8)		2 (2.0)
*> 1*		2 (0.7)		2 (1.2)		0 (0.0)
	**All individuals**		**HBM individuals**		**Relatives without HBM**	
	**Total** ***N***	**Median (IQR)**	**Total** ***N***	**Median (IQR)**	**Total** ***N***	**Median (IQR)**
WOMAC at follow-up						
*Pain*	145	0 (0, 25)	92	10 (0, 35)	53	0 (0, 15)
*Function*	145	3.6 (0, 25)	92	10.7 (0, 30.4)	53	0 (0, 14.3)
	**Total** ***N***	***N*** **(%)**	**Total** ***N***	***N*** **(%)**	**Total** ***N***	***N*** **(%)**
Hip replacement (self-reported)	145	16 (11.0)	92	13 (14.1)	53	3 (5.7)

*Abbreviations*: *HBM* high bone mass, *OA* osteoarthritis, *JSN* joint space narrowing, *WOMAC* Western Ontario and McMaster Universities Osteoarthritis Index

### Combined incidence and progression of radiographic hip OA sub-phenotypes

Of the total population, 28.8% hips had at least one osteophyte at baseline, rising to 41.5% at follow-up (Table 3). JSN was much less prevalent at baseline and follow-up (11.3% and 12.4%, respectively). In unadjusted analyses, we found evidence that individuals with HBM experienced greater changes in both osteophyte and JSN scores than individuals without HBM (*β*_osteophyte_ = 0.30 [0.05, 0.54], *p* = 0.019 and *β*_JSN_ = 0.09 [0.01, 0.16], *p* = 0.019, *β* reflects the difference in the mean change in osteophyte/JSN score between those with and without HBM). These associations persisted after adjustment for age, sex, follow-up time, baseline score, height and TBFM (model 3) (Fig. 2).

### HBM and clinical features of hip OA

**Fig. 3:**
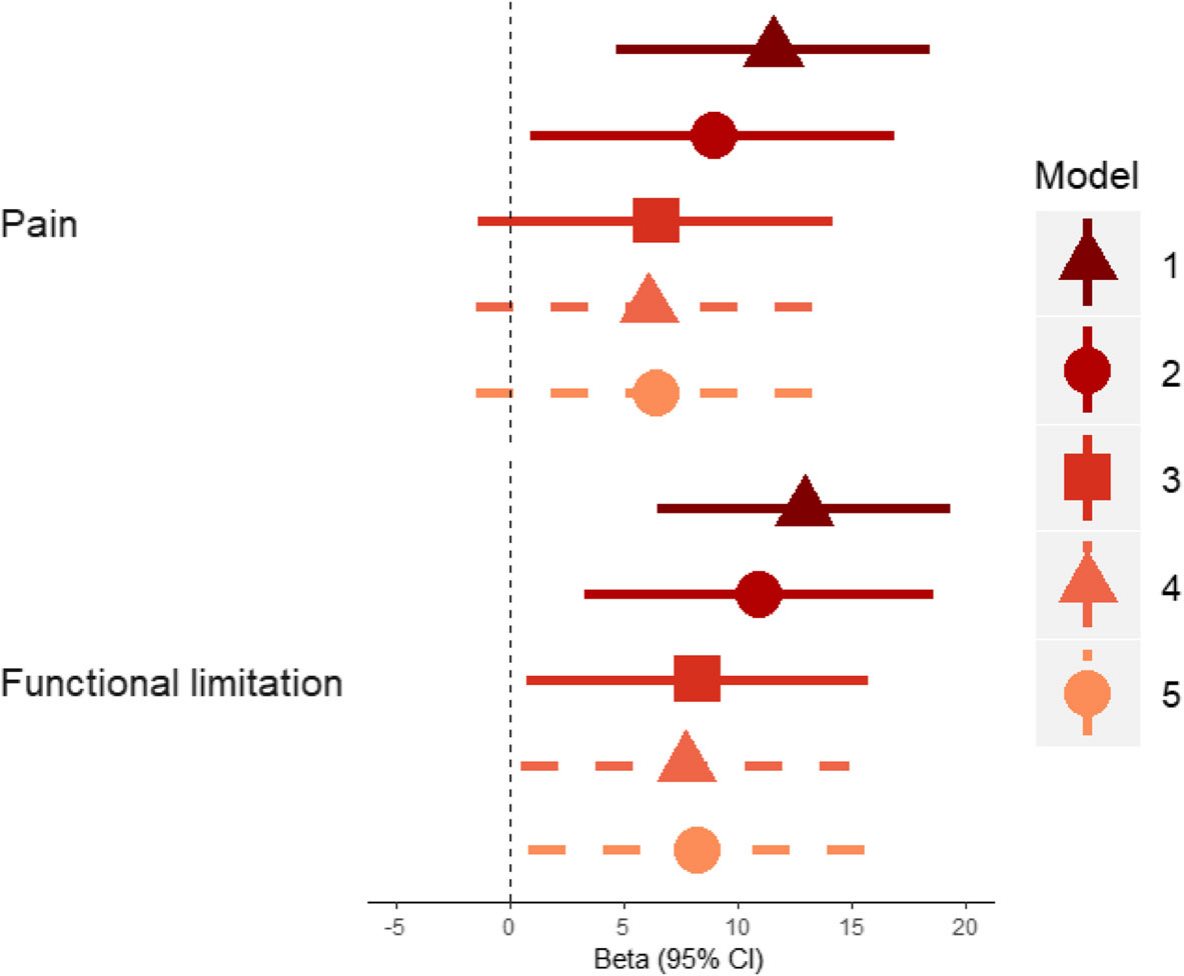
Associations between HBM status and WOMAC pain and function sub-scale scores. Points represent the mean difference in WOMAC scores between individuals with HBM and relatives/spouses without HBM. Person-level analysis, accounting for clustering in families. Follow-up osteophyte and JSN score is the highest of the two hips. Model 1: unadjusted; model 2: adjusted for age and sex; model 3: adjusted for age, sex, height and total body fat mass; model 4: model 3 plus osteophyte severity at follow-up, model 5: model 3 plus JSN severity at follow-up. *N* = 127

HBM was associated with 12-point [95% CI 5, 18] higher WOMAC pain scores and 13-point [7, 19] higher function scores in unadjusted analyses. Adjustment for age, sex, height and TBFM attenuated these relationships by approximately one-third to one-half (*β*_pain_ = 6.4 [− 1.4, 14.2], *p* = 0.105 and *β*_function_ = 8.3 [0.7, 15.8], *p* = 0.032, *β* represents the difference in mean WOMAC score between those with and without HBM). Further adjustment for osteophyte or JSN score at follow-up did not appear to explain these relationships (Fig. 3). There was some weak evidence supporting an increased odds of self-reported hip replacement in individuals with HBM who completed the follow-up questionnaire, compared to those without HBM (age, sex, height and TBFM-adjusted OR = 4.27 [0.94, 19.5], *p* = 0.061, *N* = 148).

### Sensitivity analyses

Including six individuals with an incident THR and a Croft score < 3 at baseline in the analysis of incident OA did not alter conclusions drawn. Neither did removing 10 hips from individuals who visited a different study site for follow-up radiographs, removing hips from individuals with DXA positioning errors, nor additional adjustment for TB DXA artefact. Conclusions were unchanged when performing a person-level analysis accounting for within-family clustering, although CIs were wider due to the reduced sample size (Supplementary Figure 1). Additional adjustment for the FN area (as a measure of bone size) marginally attenuated effect estimates (*β*_osteophyte_ = 0.26 [0.01, 0.52] to 0.21[− 0.02, 0.44] and *β*_JSN_ = 0.08 [0.01, 0.16] to 0.07 [4.74 × 10^−3^, 0.13]). Conclusions were unchanged when using a Poisson model, although the association between HBM and change in JSN score was stronger than the association with change in osteophyte score; however, it should be noted that these analyses may be biased by the need to recode negative values as zero.

## Discussion

We have found evidence for increased osteophyte development (i.e. incidence and/or progression) and JSN at the hip, over an average of 8 years in individuals with HBM, compared to their relatives without HBM. Radiographic JSN is thought to indirectly reflect cartilage loss [34]. Furthermore, individuals with HBM have more hip pain and limitation of function in their daily activities, which adds further evidence for increased OA severity in this population. Low statistical power limited our ability to draw strong conclusions about the relationship between HBM and overall incident OA, based on the Croft score.

Few studies have determined the association between BMD and hip OA incidence or progression. Bergink et al. observed a relationship between FN-BMD and both hip OA incidence and progression in the Rotterdam study population [14]. We have extended these findings by determining the relationship between high BMD and the incidence and/or progression of individual radiographic sub-phenotypes. Barbour et al. identified weak evidence for worsening osteophytes with increasing BMD in JoCo, but no evidence for a relationship with JSN progression [13], which is inconsistent with our observed (albeit weak) relationship between HBM and change in JSN score. Hochberg et al. identified a dose-response relationship between BMD and subsequent incidence of OA in SOF [15]. However, this relationship was no longer present when defining incidence based on JSN alone. In our analyses, whilst we did not observe strong evidence for an association between HBM and incident hip OA, possibly due to low numbers, the direction of effect was consistent with previous findings.

Our observed relationship between HBM and hip pain is consistent with studies of population-based cohorts, which have identified an increased BMD in those reporting hip pain [4]. The severity of OA sub-phenotypes did not appear to explain the relationship between HBM and hip pain or functional limitations, suggesting that HBM individuals have an increased risk of clinical OA independent of radiographic severity. The WOMAC questionnaire measures pain over the past 48 h, which may explain why radiographic OA severity did not explain current pain, as pain could increase during stages of rapid OA progression not captured by radiographs [35]. An analysis of the Framingham and OA Initiative populations found that fewer than 25% of individuals with radiographic hip OA reported hip pain, and fewer than 20% reporting hip pain had radiographic hip OA [36]. It is possible that increased pain and functional limitation in the HBM population could reflect other conditions of the hip, such as bursitis [37] or features of a mild skeletal dysplasia, or inflammation not detected on the radiograph.

Increased TBFM in the HBM population [32] did not appear to explain the relationship between HBM and change in radiographic OA sub-phenotypes. Adjustment for the FN area, as a measure of bone size, only explained a small proportion of the relationship. Unfortunately, we do not have measures of FN width, a reported risk factor for hip OA progression [38]. It is plausible that HBM individuals would have greater FN width due to greater bone mass meaning measures of FN area may not equate to FN width in this population. Another factor which may mediate the relationship between HBM and development of hip OA sub-phenotypes is differences in hip shape. HBM individuals more commonly have features of cam-type deformity (i.e. larger femoral head size and reduced sphericity) compared to their relatives without HBM [39]. Evidence suggests that cam-type deformities are a risk factor for end-stage hip OA and hence potentially for hip OA progression [40, 41].

Although HBM is likely to be caused by the polygenic inheritance of multiple BMD loci [12], or the monogenic inheritance of rare variants [42], indicating that HBM precedes OA development, we cannot rule out the possibility that biological pleiotropy, rather than a causal effect, explains our results. We have previously identified an increased prevalence of pelvic enthesophytes in the HBM population, leading to the hypothesis that HBM individuals may have a genetic predisposition to form extra bone [43]. We observed a stronger effect size for the relationship between HBM and change in hip osteophyte score, than we did for that between HBM and change in hip JSN score, which further suggests a ‘bone-forming’ phenotype in this population. Further evidence for pleiotropy was provided by Hackinger et al. who found weak evidence for a genetic correlation between hip OA and LS-BMD, but not hip OA and FN-BMD [44]. By performing a cross-phenotype meta-analysis between overall OA and LS-BMD, the authors identified novel loci in the *SMAD3* gene [44]. SMAD3 is part of the transforming growth factor β (TGFβ) signalling pathway, regulating osteoblast differentiation and thus bone formation. The first discovered hip OA locus, growth differentiation factor-5 (*GDF5*), is a ligand for this pathway [45], suggesting that this pathway contributes to both BMD and hip OA.

### Strengths and limitations

The HBM study constitutes the largest population of individuals with extreme, unexplained, generalised HBM [11]. We analysed change in OA sub-phenotypes separately, which allowed us to detect the stronger relationship with osteophyte development compared to change in JSN. We analysed change in osteophytes and JSN as continuous measures, increasing statistical power to detect associations and reducing the possibility of a ceiling effect by increasing the range of possible values from 0 to 6 for JSN and 0 to 10 for osteophytes and eliminating the possibility of selection bias in a case-only analysis.

The method of identifying individuals from NHS DXA databases ascertained a predominantly female and older population such that a relatively large proportion were unable to be followed up after 8 years, due to death or poor health. Hence, there was a lower baseline prevalence of radiographic hip OA in the population able to be followed up, meaning we had limited power to assess hip OA incidence and progression based on the overall Croft score. The baseline cross-sectional study was powered to determine if the odds of OA differed between HBM individuals and their relatives with an expected recruitment of 200 cases and 200 controls [10]. However, loss-to-follow-up over 8 years reduced our sample size and a retrospective power calculation for the analyses presented here showed that we had approximately 65% power to detect the change in osteophyte and JSN scores reported here and lower power to detect a difference in proportion of incident hip OA between HBM individuals and their non-HBM relatives. Radiographs and DXA scans were performed using standard protocols at each centre but were not standardised across centres. However, as 97% of individuals re-attended the same centre for follow-up, this is unlikely to affect our measures for *change* in radiographic features. Furthermore, measuring change in sub-phenotype variables did not separate hip OA sub-phenotype progression from incidence since these results had to be pooled to optimise sample size. As baseline and follow-up radiographs were not read as pairs, we did observe a few negative scores for change in osteophytes (8%) and change in JSN (1.5%), which were included in analyses, because removing these values as ‘measurement error’ would have biased results as there was likely to have been the same proportion of measurement error overinflating change, for which we would not have been able to account (hence the reasoning for not basing conclusions on the Poisson analysis). Radiographic grading of OA sub-phenotypes is subjective, which we limited using an established atlas [20], although our intra-rater and inter-rater reliability were low for a few variables, attenuating the conclusions we can draw from this analysis. As the reader was blinded to timepoint, it is unlikely that radiographic features were systematically under-graded at baseline and over-graded at follow-up, meaning measurement error is unlikely to explain our results. WOMAC scores were only collected at follow-up, and therefore, we cannot draw conclusions about the relationship between HBM and symptomatic OA *progression*. Finally, as HBM individuals represent a rare and extreme tail of the BMD distribution, findings may not be generalisable to the wider population.

## Conclusions

We have found evidence for associations between HBM and worsening of radiographic sub-phenotypes of hip OA over 8 years. We further provide evidence for greater symptoms of OA in HBM individuals. These associations are independent of the elevated fat mass observed in HBM individuals. Further genetic analyses are planned to determine the BMI-independent causal role of BMD in hip OA progression and to identify the underlying biological pathways explaining these associations.

## Supplementary Information

**Additional file 1: Supplementary Table 1. **Baseline characteristics for those with and without follow-up data, **Supplementary Figure 1**. associations between HBM and incident OA and incident and progressive OA sub-phenotypes in person-level analyses.

## Data Availability

The datasets during and/or analysed during the current study available from the corresponding author on reasonable request.

## Abbreviations

AP: Anteroposterior
BMD: Bone mineral density
BMI: Body mass index
CIs: Confidence intervals
DXA: Dual-energy X-ray absorptiometry
FN: Femoral neck
GEE: Generalised estimating equations
HBM: High bone mass
JoCo: Johnston County Osteoarthritis Project
JSN: Joint space narrowing
LS: Lumbar spine
MrOS: Study of Osteoporotic Fractures in Men
OA: Osteoarthritis
REC: Research Ethics Committee
sBMD: Standardised bone mineral density
SOF: Study of Osteoporotic Fractures
TB: Total body
TBFM: Total body fat mass
TH: Total hip
THR: Total hip replacement
vBMD: Volumetric bone mineral density
