# Supplementary material for: Increased development of radiographic hip osteoarthritis in individuals with high bone mass: a prospective cohort study
Source: Arthritis Res Ther. 2021 Jan 6;23:4. doi: 10.1186/s13075-020-02371-0 (PMC7788917; doi:10.1186/s13075-020-02371-0)
Supplement: Supplementary file 1 — Additional file 1: Supplementary Table 1. Baseline characteristics for those with and without follow-up data, Supplementary Figure 1. associations between HBM and incident OA and incident and progressive OA sub-phenotypes in person-level analyses. [file 13075_2020_2371_MOESM1_ESM.pdf]

Supplementary material

Increased development of radiographic hip osteoarthritis in individuals with High Bone Mass: a prospective cohort study

April Hartley[1,2*], Sarah A Hardcastle[1,3], Monika Frysz[1,2], Jon Parkinson[4], Lavinia Paternoster[2], Eugene McCloskey[5,6,7], Kenneth ES Poole[8], Muhammad K Javaid[9], Mo Aye[10], Katie Moss[11], Martin Williams[12], Jon H Tobias[1,2], Celia L Gregson[1]

1 Musculoskeletal Research Unit, Translational Health Sciences, Bristol Medical School, University of Bristol, Bristol, UK

2 MRC Integrative Epidemiology Unit, Population Health Sciences, Bristol Medical School, University of Bristol, Bristol, UK

3 Royal National Hospital for Rheumatic Diseases, Royal United Hospitals Bath NHS Foundation Trust, Bath, UK

4 Division of Informatics, Imaging & Data Sciences, Faculty of Medical and Human Sciences, University of Manchester, Manchester, UK

5 Academic Unit of Bone Metabolism, Department of Oncology and Metabolism, The Mellanby Centre For Bone Research, University of Sheffield, Sheffield, UK

6 Centre for Metabolic Diseases, University of Sheffield Medical School, Sheffield, UK

7 Centre for Integrated Research into Musculoskeletal Ageing, University of Sheffield Medical School, Sheffield, UK

8 Cambridge NIHR Biomedical Research Centre and the Wellcome Trust Clinical Research Facility, Cambridge

9 Nuffield Department of Orthopaedics, Rheumatology and Musculoskeletal Sciences, University of Oxford, Oxford, UK

10 Department of Diabetes, Endocrinology and Metabolism, Hull and East Yorkshire Hospitals NHS Trust, Hull, UK

11 Centre for Rheumatology, St George's Hospital, St George's Healthcare NHS Trust, London, UK

12 Department of Radiology, Southmead Hospital, North Bristol NHS Trust, Bristol UK

Supplementary Table 1: Baseline characteristics for those with and without follow-up data

	All<br><br>N=363	With follow-up data<br>N=145	Without follow-up data<br>N=218	*p* value for difference
		**N (%)**		
HBM cases	237 (65.3)	92 (63.5)	145 (66.5)	0.548
Female	240 (66.1)	105 (72.4)	135 (61.9)	0.039
Postmenopausal	197 (84.2)	81 (77.1)	116 (89.9)	0.008
History of smoking	208 (58.8)	74 (51.0)	134 (64.1)	0.014
Alcohol consumption				
None	79 (22.3)	25 (17.2)	54 (25.7)	
Occasional	41 (11.6)	17 (11.7)	24 (11.4)	0.154
Regular	185 (52.1)	85 (58.6)	100 (47.6)	
Heavy	50 (14.1)	18 (12.4)	32 (15.2)	
Physical activity category				
Low	58 (18.1)	16 (11.4)	42 (23.2)	0.009
Moderate	112 (34.9)	47 (33.6)	65 (35.9)	
High	151 (47.0)	77 (55.0)	74 (40.9)	
Hip OA (Croft$\geq$3)	87 (27.5)	28 (19.9)	59 (33.7)	0.006
Any hip osteophyte	248 (77.5)	102 (71.3)	146 (82.5)	0.017
Moderate hip osteophyte	82 (26.0)	30 (21.6)	52 (29.6)	0.110
Hip JSN	98 (30.8)	36 (25.5)	62 (35.0)	0.068
		**Mean (SD)**		
Age, years	62.8 (12.0)	59.6 (10.2)	64.9 (12.6)	<0.001
Height, cm	168.1 (9.7)	167.6 (9.5)	168.4 (9.8)	0.476
Weight, kg	83.4 (16.5)	82.1 (16.9)	84.2 (16.1)	0.222
L1 Z-score	2.7 (2.1)	2.5 (2.0)	2.9 (2.1)	0.069
Max total hip Z-score	2.1 (1.5)	2.1 (1.5)	2.1 (1.5)	0.953

Abbreviations: HBM: High Bone Mass; OA: Osteoarthritis; JSN: Joint Space Narrowing.

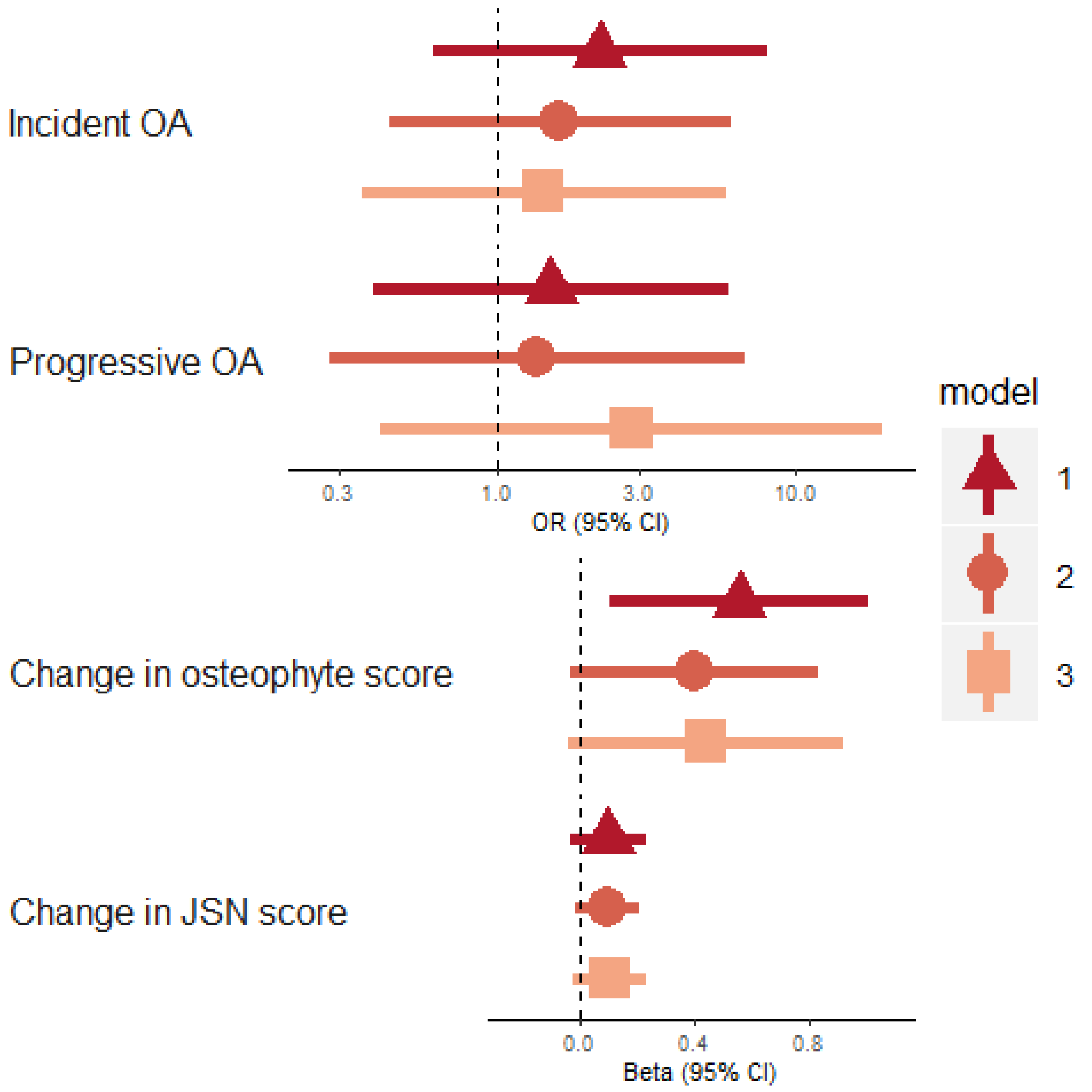

Supplementary Figure 1: associations between HBM and incident OA and incident and progressive OA sub-phenotypes in person-level analyses

Points for continuous outcomes represent the difference in mean outcome between individuals with and without HBM (For example, a beta of 1 for change in osteophyte score would represent a 1 point greater increase in summed osteophyte score, which is the equivalent of the appearance of one additional osteophyte over 8 years or the increase in size of an osteophyte already present). Points for binary outcomes represent the odds ratio for individuals with HBM compared to their relatives with normal BMD.

Model 1: unadjusted, Model 2: adjusted for age, sex and follow-up time (plus baseline score for continuous outcomes), Model 3: adjusted for age, sex, follow-up time, height and TBFM (plus baseline score for continuous outcomes)

$N_{\text{incident OA}}$=121; $N_{\text{Progressive OA}}$=54; $N_{\text{continuous outcomes}}$=126

Abbreviations: OA: Osteoarthritis; JSN: joint space narrowing
